# A Group Decision Framework with Intuitionistic Preference Relations and Its Application to Low Carbon Supplier Selection

**DOI:** 10.3390/ijerph13090923

**Published:** 2016-09-19

**Authors:** Xiayu Tong, Zhou-Jing Wang

**Affiliations:** 1 School of Management, Zhejiang University of Finance & Economics, Hangzhou 310018, China; txiayu@zufe.edu.cn; 2 School of Information, Zhejiang University of Finance & Economics, Hangzhou 310018, China

**Keywords:** intuitionistic preference relation, consistency, induced intuitionistic ordered weighted averaging operator, group decision making, low carbon supplier selection

## Abstract

This article develops a group decision framework with intuitionistic preference relations. An approach is first devised to rectify an inconsistent intuitionistic preference relation to derive an additive consistent one. A new aggregation operator, the so-called induced intuitionistic ordered weighted averaging (IIOWA) operator, is proposed to aggregate individual intuitionistic fuzzy judgments. By using the mean absolute deviation between the original and rectified intuitionistic preference relations as an order inducing variable, the rectified consistent intuitionistic preference relations are aggregated into a collective preference relation. This treatment is presumably able to assign different weights to different decision-makers’ judgments based on the quality of their inputs (in terms of consistency of their original judgments). A solution procedure is then developed for tackling group decision problems with intuitionistic preference relations. A low carbon supplier selection case study is developed to illustrate how to apply the proposed decision model in practice.

## 1. Introduction

In recent years, increasingly frequent red alerts of hazardous smog in China have created significant concerns for public health and the unsustainability of its current economic development modes [1]. Rapid increase of carbon emissions has caused climate change and resulted in global warming in the past decades. This challenge has prompted many governments and environmentalists to take actions to curb pollution. As the largest manufacturer in the world, China’s manufacturing industry reached a total value of $2.9 trillion in 2014 alone [2]. Nowadays, manufacturers in China are facing increasing pressure to develop green technology and reduce carbon emissions [3]. In this process, a critical stage is to select low carbon suppliers. Generally speaking, supplier selection requires input from different departments within the organization and judgments or preferences for comparing different suppliers are often vague and uncertain. To address this important issue, this paper first proposes a general framework to handle group decision problems where decision-makers’ (DMs’) preferences are provided by intuitionistic fuzzy judgments and the DMs’ weights are unknown. The proposed approach is then applied to a case study concerning low carbon supplier selection problems.

Since Atanassov [4] extended the fuzzy set theory to intuitionistic fuzzy sets (IFSs) by introducing both membership and non-membership functions, IFSs and their applications to decision modeling have received increasing attention from researchers. Thanks to their flexibility in characterizing inherent vagueness and hesitancy in the human decision making process, IFSs have been extensively studied in the area of multiple attribute decision making [5,6,7,8,9]. A common multiattribute decision framework is to take pairwise comparison preference relations as basic input. Along this line, Szmidt and Kacprzyk [10] represent an intuitionistic preference relation as a combination of a fuzzy preference matrix and a hesitancy matrix, and investigate how to aggregate individual DMs’ intuitionistic preference relations in a group decision making (GDM) setting. Xu [11] employs intuitionistic fuzzy numbers (IFNs) to describe DMs’ pairwise judgments, thereby defining intuitionistic preference relations. Subsequently, Xu [12] introduces a compatibility measure between intuitionistic preference relations, and applies it to develop a consensus reaching procedure in GDM. When a pairwise comparison matrix is employed to depict a DM’s preference, a critical issue is the consistency of the preference relation [13]. In the context of intuitionistic preference relations, different consistency definitions have been proposed [14,15]. For instance, based on IFNs operations [16], Xu [11] defines multiplicative consistent intuitionistic preference relations, and introduces an intuitionistic fuzzy weighted averaging (IFWA) operator to develop an approach to GDM with intuitionistic preference relations, in which the weights of DMs are known. Subsequently, Xu et al. [17] point out the deficiency of the multiplicative transitivity condition in Xu [11]. Motivated by the multiplicative consistency equivalence formula of fuzzy preference relations, Xu et al. [17] furnish a new multiplicative consistency definition for intuitionistic preference relations. On the other hand, Gong et al. [18] introduce an additive consistency definition for intuitionistic preference relations and investigate how to derive priority weights by establishing a goal programming model and a least squares model. Wang [19] shows that the additive consistency in Gong et al. [18] is defined in an indirect manner, and the matrix based on the consistency transformation equations therein may not necessarily be an intuitionistic preference relation. As such, Wang [19] introduces a new additive consistency definition by employing membership and non-membership of the DM’s intuitionistic judgments and establishes goal programming models to derive intuitionistic fuzzy weights.

In GDM, an important topic is to aggregate individual preference values into a group preference [20,21,22,23]. The ordered weighted averaging (OWA) [24] and the induced ordered weighted averaging (IOWA) [25] operators have been extended to situations where decision inputs are provided as IFNs. These extensions range from the intuitionistic fuzzy ordered weighted averaging (IFOWA) operator [16], to the intuitionistic fuzzy ordered weighted geometric (IFOWG) operator [26], the induced intuitionistic fuzzy ordered weighted geometric (IIFOWG) operator [27], the induced intuitionistic fuzzy ordered weighted averaging (I-IFOWA) operator [28], and the induced generalized intuitionistic fuzzy ordered weighted averaging (IG-IFOWA) operator [29]. These aggregation operators take different operational laws to treat membership and non-membership information. The drawback of this treatment is that these aggregation operators do not necessarily possess the desired monotonic property as per the ranking based on the score and accuracy functions. The implication is that, even if all individual intuitionistic preference relations are consistent, the aggregated one may not be consistent. Another drawback is that the complement property (see Theorem 5 in Section 4) will not be maintained by employing these aggregation operators, implying that it is difficult to use them for aggregating individual intuitionistic preference relations. On the other hand, in the process of GDM, it is often the case that the intuitionistic preference relations provided by the DMs are inconsistent. To obtain a reasonable decision result, it is necessary to first rectify consistency of these intuitionistic preference relations. By employing the additive consistency definition introduced by Wang [19], this paper puts forward a consistency rectification framework to tackle GDM problems with intuitionistic preference relations. The proposed procedure consists of three stages: (1) Rectification of individual inconsistent intuitionistic preference relations; (2) Aggregation of the rectified consistent intuitionistic preference relations; (3) Ranking of alternatives based on aggregated preference values. More specifically, an innovative approach is first proposed to rectify any inconsistent intuitionistic preference relation. An induced intuitionistic ordered weighted averaging (IIOWA) operator is then developed to aggregate individual IFNs, in which the same weighted averaging scheme is applied to both the membership and non-membership of IFNs. Subsequently, the mean absolute deviation (MAD) between the original and rectified intuitionistic preference relations is adopted as an order inducing variable of the IIOWA operator to aggregate the rectified consistent intuitionistic preference relations. A key objective of this treatment is to assign different weights to different DMs’ judgments as per the quality of the inputs (in terms of consistency of the DMs’ original judgments). Finally, a solution procedure is summarized for handling GDM with intuitionistic preference relations and applied to a low carbon supplier selection problem to illustrate its applicability and effectiveness.

The remainder of this paper is organized as follows. Section 2 furnishes basic concepts of IFSs and additive consistent intuitionistic preference relations. Section 3 proposes an approach to rectifying inconsistent intuitionistic preference relations. In Section 4, a new aggregation operator, IIOWA, is developed to aggregate intuitionistic preference values, followed by a procedure for solving GDM problems with intuitionistic preference relations. A low carbon supplier selection example is presented to illustrate the proposed approach in Section 5. Concluding remarks are offered in Section 6.

## 2. Preliminaries

This section presents basic concepts of IFSs and additive consistent intuitionistic preference relations. The aim is to put future discussions in a proper context.

By introducing membership and non-membership functions, Atanassov [4] put forward the notion of IFSs as follows.

**Definition 1.**  *Let a nonempty set Z be fixed, an IFS A in Z can be defined as*

(1) $$A=\{<z,\mu_{A}(z),\nu_{A}(z)>|z\in Z\}$$

*where* $0\leq \mu_{A}(z)\leq 1,0\leq \nu_{A}(z)\leq 1,\mu_{A}(z)+\nu_{A}(z)\leq 1$, ∀z∈Z.

$\mu_{A}(z)$ and $\nu_{A}(z)$ denote the membership and non-membership degree of element *z* to set *A*, respectively. In addition, $\pi_{A}(z)=1-\mu_{A}(z)-\nu_{A}(z)$ is called the hesitancy degree of *z* to *A*. Obviously, $0\leq \pi_{A}(z)\leq 1$ for every z∈Z.

For a given *z* and IFS A, the pair $\left(\mu_{A}(z),\nu_{A}(z)\right)$ is referred to as an IFN [26]. For notational and computational convenience, an IFN is often denoted by $\tilde{\alpha }=(\mu ,v)$, where 0≤μ,v≤1 and μ+v≤1.

To compare two IFNs, a score function is defined by Chen and Tan [30] as

(2) $$S(\tilde{\alpha })=\mu -v$$

and subsequently, an accuracy function is defined by Hong and Choi [31] as

(3) $$H(\tilde{\alpha })=\mu +v$$

It is obvious that $-1\leq S(\tilde{\alpha })\leq 1$, $0\leq H(\tilde{\alpha })\leq 1$, and the hesitancy degree of $\tilde{\alpha }$ can be computed as

(4) $$\pi (\tilde{\alpha })=1-H(\tilde{\alpha })$$

The higher the accuracy degree of $\tilde{\alpha }$, the lower its hesitancy degree. If $\pi (\tilde{\alpha })=0$, then ν=1−μ, indicating that the IFN $\tilde{\alpha }$ is reduced to a fuzzy number μ.

Let ${\tilde{\alpha }}_{1}=(\mu_{1},v_{1})$ and ${\tilde{\alpha }}_{2}=(\mu_{2},v_{2})$ be any two IFNs, based on the aforesaid score and accuracy functions, Xu and Yager [26] propose a prioritized comparison method for two IFNs as follows:

If $S({\tilde{\alpha }}_{1})<S({\tilde{\alpha }}_{2})$, then ${\tilde{\alpha }}_{1}$ is smaller than ${\tilde{\alpha }}_{2}$, which is denoted by ${\tilde{\alpha }}_{1}<{\tilde{\alpha }}_{2}$.
If $S({\tilde{\alpha }}_{1})=S({\tilde{\alpha }}_{2})$, then
  if $H({\tilde{\alpha }}_{1})<H({\tilde{\alpha }}_{2})$, then ${\tilde{\alpha }}_{1}$ is smaller than ${\tilde{\alpha }}_{2}$, which is denoted by ${\tilde{\alpha }}_{1}<{\tilde{\alpha }}_{2}$;
  if $H({\tilde{\alpha }}_{1})=H({\tilde{\alpha }}_{2})$, then ${\tilde{\alpha }}_{1}$ and ${\tilde{\alpha }}_{2}$ are equal, which is denoted by ${\tilde{\alpha }}_{1}={\tilde{\alpha }}_{2}$.

The aforesaid comparison method for any two IFNs implies that ${\tilde{\alpha }}_{1}\leq {\tilde{\alpha }}_{2}$ if and only if (i) $S({\tilde{\alpha }}_{1})<S({\tilde{\alpha }}_{2})$ or (ii) $S({\tilde{\alpha }}_{1})=S({\tilde{\alpha }}_{2})$ and $H({\tilde{\alpha }}_{1})\leq H({\tilde{\alpha }}_{2})$.

To express hesitancy and uncertainty in DMs’ pairwise judgments, Xu [11] introduces the concept of intuitionistic preference relations.

Let $X=\{x_{1},x_{2},...,x_{n}\}$ be a discrete set of decision alternatives. An intuitionistic preference relation on $X=\{x_{1},x_{2},...,x_{n}\}$ is characterized by a matrix $\tilde{R}={\left({\tilde{r}}_{ij}\right)}_{n\times n}\subset X\times X$, where ${\tilde{r}}_{ij}=(\mu_{ij},v_{ij})$ denotes an intuitionistic preference value of alternative $x_{i}$ over $x_{j}$ such that

(5) $$0\leq \mu_{ij}\leq 1,0\leq v_{ij}\leq 1,\mu_{ij}+v_{ij}\leq 1,\mu_{ij}=v_{ji},v_{ij}=\mu_{ji},\mu_{ii}=v_{ii}=0.5,i,j=1,2,\ldots ,n$$

It is clear that each preference value ${\tilde{r}}_{ij}=(\mu_{ij},v_{ij})$ in $\tilde{R}$ is an IFN. $\mu_{ij}$ and $v_{ij}$ indicate, respectively, membership and non-membership degrees to which alternative $x_{i}$ is superior to $x_{j}$.

Due to $\mu_{ij}=v_{ji}$ in $\tilde{R}$ for all *i*, *j* = 1, 2, …, *n*, Wang [19] defines the following additive consistent intuitionistic preference relations by directly using the membership degrees in the judgment matrix.

**Definition 2.**  *An intuitionistic preference relation* $\tilde{R}={\left({\tilde{r}}_{ij}\right)}_{n\times n}$ *with* ${\tilde{r}}_{ij}=(\mu_{ij},v_{ij})$ *is additive consistent if the following additive transitivity is satisfied.*

(6) $$\mu_{ij}+\mu_{jk}+\mu_{ki}=\mu_{kj}+\mu_{ji}+\mu_{ik}\;\text{for all}\;i,j,k=1,2,...,n$$

Since $\mu_{ij}=v_{ji},v_{ij}=\mu_{ji}$ for all *i*, *j* = 1, 2, …, *n*, it follows from (6) that

(7) $$v_{ij}+v_{jk}+v_{ki}=v_{kj}+v_{ji}+v_{ik}\;\text{for all}\;i,j,k=1,2,...,n$$

Based on Definition 2 and the score function S(.), Wang [19] establishes the following result to judge the additive consistency of an intuitionistic preference relation.

**Lemma 1.**  *An intuitionistic preference relation* $\tilde{R}={\left({\tilde{r}}_{ij}\right)}_{n\times n}$ *is additive consistent if and only if*

(8) $$S({\tilde{r}}_{ij})=S({\tilde{r}}_{ik})-S({\tilde{r}}_{jk})\;\text{for all}\;i,j,k=1,2,...,n.$$

## 3. Rectification of Inconsistent Intuitionistic Preference Relations

According to Lemma 1, the additive consistency of an intuitionistic preference relation $\tilde{R}$ can be verified by checking whether the scores of the intuitionistic judgments in $\tilde{R}$ satisfy (8). To derive a consistent intuitionistic preference relation from an inconsistent judgment matrix $\tilde{R}$, a sensible approach is to adjust the scores of some elements in $\tilde{R}$. On the other hand, it is clear from (2) that different IFNs may possess an identical score value as long as they have the same difference between their corresponding membership and non-membership degrees. So, a critical issue is how to properly adjust these score values in the rectification process. To avoid excessive distortion of the DM’s original judgment, it is desirable that the rectified IFNs should be as close to the original IFNs in $\tilde{R}$ as possible. As per (2) and (4), one can see that an IFN can be uniquely determined by its score and hesitancy values. Given these considerations, the rectified IFNs are selected to maintain the original hesitancy values. If an additive consistent intuitionistic preference relation cannot be obtained by keeping hesitancy values constant, their hesitancy values will be scaled down proportionally in the rectification process.

For a given intuitionistic preference relation $\tilde{R}={\left({\tilde{r}}_{ij}\right)}_{n\times n}$, let

(9) $${\hat{\mu }}_{ij}=\frac{1}{2n}\left(\sum_{l=1}^{n}S({\tilde{r}}_{il})-\sum_{l=1}^{n}S({\tilde{r}}_{jl})\right)+0.5\left(1-\pi ({\tilde{r}}_{ij})\right)\;i,j=1,2,...,n$$

(10) $${\hat{v}}_{ij}=\frac{1}{2n}\left(\sum_{l=1}^{n}S({\tilde{r}}_{jl})-\sum_{l=1}^{n}S({\tilde{r}}_{il})\right)+0.5\left(1-\pi ({\tilde{r}}_{ij})\right)\;i,j=1,2,...,n$$

Then, we have the following result.

**Theorem 1.**  *Let* $\tilde{R}={\left({\tilde{r}}_{ij}\right)}_{n\times n}$ *with* ${\tilde{r}}_{ij}=(\mu_{ij},v_{ij})$ *be an intuitionistic preference relation, and* ${\hat{\mu }}_{ij}$ *and* ${\hat{v}}_{ij}$ (i,j=1,2,...,n) *be defined by (9) and (10), respectively, then*

*(i)*  ${\hat{\mu }}_{ii}={\hat{v}}_{ii}=0.5$, ∀i=1,2,...,n.
*(ii)*  $0\leq {\hat{\mu }}_{ij}+{\hat{v}}_{ij}\leq 1$, ∀i,j=1,2,...,n.
*(iii)*  ${\hat{\mu }}_{ij}={\hat{v}}_{ji}$ *and* ${\hat{v}}_{ij}={\hat{\mu }}_{ji}$, ∀i=1,2,...,n.
*(iv)*  ${\hat{\mu }}_{ij}+{\hat{v}}_{ij}=\mu_{ij}+v_{ij}$, ∀i,j=1,2,...,n.
*(v)*  ${\hat{\mu }}_{ij}+{\hat{\mu }}_{jk}+{\hat{\mu }}_{ki}={\hat{\mu }}_{kj}+{\hat{\mu }}_{ji}+{\hat{\mu }}_{ik}$, ∀i,j,k=1,2,...,n.
*(vi)*  ${\hat{v}}_{ij}+{\hat{v}}_{jk}+{\hat{v}}_{ki}={\hat{v}}_{kj}+{\hat{v}}_{ji}+{\hat{v}}_{ik}$, ∀i,j,k=1,2,...,n.

**Proof.**  Since $\tilde{R}$ is an intuitionistic preference relation, as per (4) and (5), we have $\pi ({\tilde{r}}_{ii})=\pi ((0.5,0.5))=1-0.5-0.5=0$, $0\leq \pi ({\tilde{r}}_{ij})=1-\mu_{ij}-v_{ij}\leq 1$, $\pi ({\tilde{r}}_{ij})=1-\mu_{ij}-v_{ij}=1-\;v_{ji}-\mu_{ji}=\pi ({\tilde{r}}_{ji})$ and $1-\pi ({\tilde{r}}_{ij})=\mu_{ij}+v_{ij}$ for all i,j=1,2,...,n. Therefore, (i)–(iv) can be derived from (9) and (10).

As $\pi ({\tilde{r}}_{ij})=\pi ({\tilde{r}}_{ji})$ for all i,j=1,2,...,n, it follows from (9) that

$$\begin{array}{l} {\hat{\mu }}_{ij}+{\hat{\mu }}_{jk}+{\hat{\mu }}_{ki}=0.5\left(1-\pi ({\tilde{r}}_{ij})\right)+0.5\left(1-\pi ({\tilde{r}}_{jk})\right)+0.5\left(1-\pi ({\tilde{r}}_{ki})\right)\\ =0.5\left(1-\pi ({\tilde{r}}_{kj})\right)+0.5\left(1-\pi ({\tilde{r}}_{ji})\right)+0.5\left(1-\pi ({\tilde{r}}_{ik})\right)={\hat{\mu }}_{kj}+{\hat{\mu }}_{ji}+{\hat{\mu }}_{ik} \end{array}$$

Similarly, from (10), we have ${\hat{v}}_{ij}+{\hat{v}}_{jk}+{\hat{v}}_{ki}={\hat{v}}_{kj}+{\hat{v}}_{ji}+{\hat{v}}_{ik}$ ∀i,j,k=1,2,...,n. ☐

Denote a matrix by $\hat{\tilde{R}}={\left({\hat{\tilde{r}}}_{ij}\right)}_{n\times n}$ with ${\hat{\tilde{r}}}_{ij}=({\hat{\mu }}_{ij},{\hat{v}}_{ij})$, where ${\hat{\mu }}_{ij}$ and ${\hat{v}}_{ij}$ (i,j=1,2,...,n) are defined by (9) and (10), respectively. From Definition 2 and Theorem 1, one can easily obtain the following corollary.

**Corollary 1.**  *If* ${\hat{\mu }}_{ij}\geq 0$ *and* ${\hat{v}}_{ij}\geq 0$ *for all* i,j=1,2,...,n*, then* $\hat{\tilde{R}}={\left({\hat{\tilde{r}}}_{ij}\right)}_{n\times n}$ *is a consistent intuitionistic preference relation and* $\pi ({\hat{\tilde{r}}}_{ij})=\pi ({\tilde{r}}_{ij})$.

**Theorem 2.**  *If* $\tilde{R}={\left({\tilde{r}}_{ij}\right)}_{n\times n}$ *is an additive consistent intuitionistic preference relation, then* $\hat{\tilde{R}}=\tilde{R}$.

**Proof.**  If $\tilde{R}$ is additive consistent, from Lemma 1, one has $S({\tilde{r}}_{ij})=S({\tilde{r}}_{ik})-S({\tilde{r}}_{jk})$ ∀*i*, *j*, *k* = 1, 2, …, *n*. Thus,

$$\frac{1}{2n}\left(\sum_{l=1}^{n}S({\tilde{r}}_{il})-\sum_{l=1}^{n}S({\tilde{r}}_{jl})\right)=\frac{1}{2n}\sum_{l=1}^{n}\left(S({\tilde{r}}_{il})-S({\tilde{r}}_{jl})\right)=\frac{1}{2n}\left(nS({\tilde{r}}_{ij})\right)=0.5S({\tilde{r}}_{ij})$$

As per (2), (4), (9), and (10), one confirms that

$${\hat{\mu }}_{ij}=0.5S({\tilde{r}}_{ij})+0.5(1-\pi ({\tilde{r}}_{ij}))=0.5(\mu_{ij}-v_{ij})+0.5(\mu_{ij}+v_{ij})=\mu_{ij}$$

$${\hat{v}}_{ij}=-0.5S({\tilde{r}}_{ij})+0.5(1-\pi ({\tilde{r}}_{ij}))=-0.5(\mu_{ij}-v_{ij})+0.5(\mu_{ij}+v_{ij})=v_{ij}$$

This proves that $\hat{\tilde{R}}=\tilde{R}$. ☐

Corollary 1 reveals that a consistent intuitionistic preference relation $\hat{\tilde{R}}$ can be derived from $\tilde{R}$ by using the Formulaes (9) and (10) provided that ${\hat{\mu }}_{ij}\geq 0$ and ${\hat{v}}_{ij}\geq 0$ for all i,j=1,2,...,n. In this case, the hesitancy degree of each IFN in $\hat{\tilde{R}}$ remains the same as that of the corresponding element in $\tilde{R}$. Theorem 2 demonstrates that $\hat{\tilde{R}}=\tilde{R}$ if the original intuitionistic preference relation $\tilde{R}$ is additive consistent. On the other hand, if $\tilde{R}$ is not consistent, Equations (9) and (10) may yield ${\hat{\mu }}_{ij}<0$, ${\hat{\mu }}_{ij}>1$, ${\hat{\nu }}_{ij}<0$, or ${\hat{\nu }}_{ij}>1$. In this case, $\hat{\tilde{R}}$ will not be an intuitionistic preference relation. To derive a consistent intuitionistic fuzzy preference relation, $\left({\hat{\mu }}_{ij},{\hat{v}}_{ij}\right)$ (*i*, *j* = 1, 2, …, *n*) in $\hat{\tilde{R}}$ have to be converted into IFNs by using a transformation function as shown below.

Let

(11) $$d=\{\begin{array}{ll} \;0, & \mathrm{if}\;{\hat{\mu }}_{ij}\geq 0,\forall i,j=1,2,...,n\\ \max\{\left|{\hat{\mu }}_{ij}\right||{\hat{\mu }}_{ij}<0,i,j=1,2,...,n\}, & \;\mathrm{otherwise} \end{array}$$

It is obvious that d≥0, and ${\hat{\mu }}_{ij}\geq -d$ ∀i,j=1,2,...,n. As per Theorem 1, one has ${\hat{v}}_{ij}={\hat{\mu }}_{ji}\geq -d,0\leq {\hat{\mu }}_{ij}+{\hat{v}}_{ij}\leq 1$ ∀i,j=1,2,...,n. Thus, $-d\leq {\hat{\mu }}_{ij}\leq 1+d$, $-d\leq {\hat{v}}_{ij}\leq 1+d$ ∀i,j=1,2,...,n, i.e., ${\hat{\mu }}_{ij}\in [-d,1+d],{\hat{v}}_{ij}\in [-d,1+d]$ ∀i,j=1,2,...,n.

As the membership and non-membership degrees of an IFN lie between 0 and 1, (0, 1) and (1, 0) are the smallest and largest IFNs, respectively. To derive an additive consistent intuitionistic preference relation from the matrix $\hat{\tilde{R}}$, a proper transformation function f:[−d,1+d]×[−d,1+d]→[0,1]×[0,1] should possess the following properties:

(i) f(−d,1+d)=(0,1).
(ii) f(1+d,−d)=(1,0).
(iii) f(0.5,0.5)=(0.5,0.5).
(iv) ${\left(f(x,y)\right)}^{c}=f\left(y,x\right)$ ∀x,y∈[−d,1+d], where ${\left(x,y\right)}^{c}$ is the complement of (x,y), i.e., ${\left(x,y\right)}^{c}=(y,x)$.
(v) ∀x,y∈[−d,1+d], if x+y≤1, then $\mu_{f}^{xy}+\nu_{f}^{xy}\leq 1$, where $\left(\mu_{f}^{xy},\nu_{f}^{xy})=f(x,y\right)$.
(vi) $\forall x_{1},x_{2},x_{3},x_{4},x_{5},x_{6},y_{1},y_{2},y_{3},y_{4},y_{5},y_{6}\in [-d,1+d]$, if $x_{1}+x_{2}+x_{3}=x_{4}+x_{5}+x_{6}$ and $y_{1}+y_{2}+y_{3}=y_{4}+y_{5}+y_{6}$, then $\mu_{f}^{x_{1}y_{1}}+\mu_{f}^{x_{2}y_{2}}+\mu_{f}^{x_{3}y_{3}}=\mu_{f}^{x_{4}y_{4}}+\mu_{f}^{x_{5}y_{5}}+\mu_{f}^{x_{6}y_{6}}$ and $v_{f}^{x_{1}y_{1}}+v_{f}^{x_{2}y_{2}}+v_{f}^{x_{3}y_{3}}=v_{f}^{x_{4}y_{4}}+v_{f}^{x_{5}y_{5}}+v_{f}^{x_{6}y_{6}}$, where $\left(\mu_{f}^{x_{k}y_{k}},v_{f}^{x_{k}y_{k}}\right)=f(x_{k},y_{k})$ *k* = 1, 2, …, 6.

Properties (i) and (ii) require the transformation function to convert the pairs (−d,1+d) and (1+d,−d) to the smallest IFN (0, 1) and the largest IFN (1, 0), respectively; (iii) ensures that an indifferent judgment (0.5, 0.5) remains after f(⋅,⋅) is applied; (iv) expects f(⋅,⋅) to maintain the complementary property under ${\left(x,y\right)}^{c}=(y,x)$; (v) guarantees that the converted values f(x,y) ∀x,y∈[−d,1+d] are IFNs if x+y≤1. The last property (vi) makes sure that the conversion procedure retains additive transitivity.

If a transformation function f(⋅,⋅) possesses the aforesaid six properties, as per Theorem 1, it can be immediately confirmed that $f\left(\hat{\tilde{R}}\right)={\left(f\left({\hat{\tilde{r}}}_{ij}\right)\right)}_{n\times n}={\left(f\left({\hat{\mu }}_{ij},{\hat{\nu }}_{ij}\right)\right)}_{n\times n}$ is an additive consistent intuitionistic preference relation.

More specifically, based on the transformation function furnished for fuzzy preference relations by Herrera-Viedma et al. [32], let

(12) $$f_{0}(x,y)=\left(\frac{x+d}{1+2d},\frac{y+d}{1+2d}\right)\;\forall x,y\in [-d,1+d]$$

Then, it is obvious that $f_{0}(\cdot ,\cdot )$ satisfies the aforesaid desired properties (i)–(iv). Since $\mu_{f_{0}}^{xy}+v_{f_{0}}^{xy}=\frac{x+d}{1+2d}+\frac{y+d}{1+2d}=\frac{x+y+2d}{1+2d}$, it is confirmed that $\mu_{f_{0}}^{xy}+v_{f_{0}}^{xy}\leq 1$ if x+y≤1. Thus, $f_{0}(\cdot ,\cdot )$ satisfies the property (v). To verify (vi), for $\forall x_{1},x_{2},x_{3},x_{4},x_{5},x_{6},y_{1},y_{2},y_{3},y_{4},y_{5},y_{6}\in [-d,1+d]$, if $x_{1}+x_{2}+x_{3}=x_{4}+x_{5}+x_{6}$ and $y_{1}+y_{2}+y_{3}=y_{4}+y_{5}+y_{6}$, then $\mu_{f_{0}}^{x_{1}y_{1}}+\mu_{f_{0}}^{x_{2}y_{2}}+\mu_{f_{0}}^{x_{3}y_{3}}=\frac{x_{1}+d}{1+2d}+\frac{x_{2}+d}{1+2d}+\frac{x_{3}+d}{1+2d}=\frac{x_{1}+x_{2}+x_{3}+3d}{1+2d}=\frac{x_{4}+x_{5}+x_{6}+3d}{1+2d}=\mu_{f_{0}}^{x_{4}y_{4}}+\mu_{f_{0}}^{x_{5}y_{5}}+\mu_{f_{0}}^{x_{6}y_{6}}$ and $v_{f_{0}}^{x_{1}y_{1}}+v_{f_{0}}^{x_{2}y_{2}}+v_{f_{0}}^{x_{3}y_{3}}=\frac{y_{1}+d}{1+2d}+\frac{y_{2}+d}{1+2d}+\frac{y_{3}+d}{1+2d}=\frac{y_{1}+y_{2}+y_{3}+3d}{1+2d}=\frac{y_{4}+y_{5}+y_{6}+3d}{1+2d}=v_{f_{0}}^{x_{4}y_{4}}+v_{f_{0}}^{x_{5}y_{5}}+v_{f_{0}}^{x_{6}y_{6}}$. Therefore, $f_{0}(\cdot ,\cdot )$ possesses the desired property (vi) as well.

By applying the transformation function $f_{0}(\cdot ,\cdot )$, ${\hat{\tilde{r}}}_{ij}$ is converted to ${\hat{\tilde{r}}}_{ij}^{\prime }$ as follows:

(13) $${\hat{\tilde{r}}}_{ij}^{\prime }=({\hat{\mu }}_{ij}^{\prime },{\hat{v}}_{ij}^{\prime })=f_{0}({\hat{\tilde{r}}}_{ij})=f_{0}({\hat{\mu }}_{ij},{\hat{v}}_{ij})=\left(\frac{{\hat{\mu }}_{ij}+d}{1+2d},\frac{{\hat{v}}_{ij}+d}{1+2d}\right)$$

where ${\hat{\tilde{r}}}_{ij}=({\hat{\mu }}_{ij},{\hat{v}}_{ij})$ is defined by (9) and (10).

**Theorem 3.**  *Let* $\tilde{R}={\left({\tilde{r}}_{ij}\right)}_{n\times n}$ *be an intuitionistic preference relation, and the elements of* ${\hat{\tilde{R}}}^{\prime }=f_{0}(\hat{\tilde{R}})={\left({\hat{\tilde{r}}}_{ij}^{\prime }\right)}_{n\times n}$ *be defined by (13), then* ${\hat{\tilde{R}}}^{\prime }$ *is an additive consistent intuitionistic preference relation and* $\pi ({\hat{\tilde{r}}}_{ij}^{\prime })=\frac{1}{1+2d}\pi ({\tilde{r}}_{ij})$.

**Proof.**  As $f_{0}(\cdot ,\cdot )$ satisfies the aforesaid six desired properties of a transformation function, it immediately follows that ${\hat{\tilde{R}}}^{\prime }$ is an additive consistent intuitionistic preference relation. As per Theorem 1, ${\hat{\mu }}_{ij}+{\hat{v}}_{ij}=\mu_{ij}+v_{ij}$ for all i,j=1,2,...,n. By (4), one has $\pi ({\hat{\tilde{r}}}_{ij}^{\prime })=1-\frac{{\hat{\mu }}_{ij}+d}{1+2d}-\frac{{\hat{v}}_{ij}+d}{1+2d}=\frac{1}{1+2d}(1-{\hat{\mu }}_{ij}-{\hat{v}}_{ij})=\frac{1}{1+2d}(1-\mu_{ij}-v_{ij})=\frac{1}{1+2d}\pi ({\tilde{r}}_{ij})$. ☐

Theorem 3 furnishes an approach to rectifying any intuitionistic preference relation $\tilde{R}={\left({\tilde{r}}_{ij}\right)}_{n\times n}$. If $\tilde{R}$ is consistent, the rectification process ends up with the same $\tilde{R}$. For an inconsistent intuitionistic preference relation $\tilde{R}$, if *d* = 0, the rectification process stops at ${\hat{\tilde{R}}}^{\prime }=\hat{\tilde{R}}$ and the hesitancy degree of each IFN in the rectified intuitionistic preference relation ${\hat{\tilde{R}}}^{\prime }$ equals that of the corresponding original judgment in $\tilde{R}$; if *d* > 0, the hesitancy degree of each IFN in $\tilde{R}$ is scaled down by a common proportion 1/(1 + 2*d*).

For an inconsistent original judgment matrix $\tilde{R}$, this additive consistency rectification process can be summarized as follows:

Step 1. Construct $\hat{\tilde{R}}={\left({\hat{\tilde{r}}}_{ij}\right)}_{n\times n}$ with ${\hat{\tilde{r}}}_{ij}=({\hat{\mu }}_{ij},{\hat{v}}_{ij})$ from $\tilde{R}$ as per (9) and (10).

Step 2. Determine the value of *d* by Equation (11). If *d* = 0, ${\hat{\tilde{R}}}^{\prime }=\hat{\tilde{R}}$ and stop; otherwise, go to Step 3.

Step 3. Calculate ${\hat{\tilde{r}}}_{ij}^{\prime }$ (*i*, *j* = 1, 2, …, *n*) to transform $\hat{\tilde{R}}$ into an additive consistent intuitionistic preference relation ${\hat{\tilde{R}}}^{\prime }={\left({\hat{\tilde{r}}}_{ij}^{\prime }\right)}_{n\times n}$ by Equation (13).

## 4. An Approach to Group Decision Making with Intuitionistic Preference Relations

### 4.1. An Induced Intuitionistic Ordered Weighted Averaging (IIOWA) Operator

Yager and Filev [25] extend the ordered weighted averaging (OWA) operator [24] to an induced ordered weighted averaging (IOWA) operator by introducing an order inducing variable as defined below.

**Definition 3.**  *An IOWA operator is a function* $IOWA:{\left(ℜ\times ℜ\right)}^{m}\to ℜ$ *defined by an associated m-dimensional weight vector* $\omega ={\left(\omega_{1},\omega_{2},...,\omega_{m}\right)}^{T}$ *such that* $\sum_{i=1}^{m}\omega_{i}=1$ *and* $\omega_{i}\in [0,1]$ *(i = 1, 2, …, m), and a set of pairs* $\left\{<I_{1},a_{1}>,<I_{2},a_{2}>,...,<I_{m},a_{m}>\right\}$*, as per the following expression:*

(14) $$IOWA_{\omega }\left(<I_{1},a_{1}>,<I_{2},a_{2}>,...,<I_{m},a_{m}>\right)=\sum_{i=1}^{m}\omega_{i}a_{\sigma (i)}$$

*where* σ *is a permutation of {1, 2, …, m} such that* $I_{\sigma (i)}\geq I_{\sigma (i+1)}$ *for i = 1, 2, …, m-1, i.e.,* $\left(a_{\sigma (1)},a_{\sigma (2)},...,a_{\sigma (m)}\right)$ *is a reordering of* $\left(a_{1},a_{2},...,a_{m}\right)$ *as per a decreasing order of all* $I_{i}$ *(i = 1, 2, …, m).*

In Definition 3, $I_{i}$ in the pair $<I_{i},a_{i}>$ is referred to as the value of an order inducing variable and $a_{i}$ as the value of an argument variable. The reordering may be generalized as an ascending order. In this case, it is necessary to distinguish between a descending IOWA operator (DIOWA) and an ascending IOWA (AIOWA) operator.

In the following, the IOWA operator is extended to accommodate situations where the input arguments are expressed as IFNs.

**Definition 4.**  *Let* $\alpha_{i}=(\mu_{i},v_{i})$ *(i = 1, 2, …, m) be m IFNs, then an induced intuitionistic ordered weighted averaging (IIOWA) operator is defined as:*

(15) $$\begin{array}{l} IIOWA_{\omega }\left(<I_{1},\alpha_{1}>,<I_{2},\alpha_{2}>,...,<I_{m},\alpha_{m}>\right)=\\ \left(IOWA_{\omega }\left(<I_{1},\mu_{1}>,<I_{2},\mu_{2}>,...,<I_{m},\mu_{m}>\right),IOWA_{\omega }\left(<I_{1},v_{1}>,<I_{2},v_{2}>,...,<I_{m},v_{m}>\right)\right)\\ =\left(\sum_{i=1}^{m}\omega_{i}\mu_{\sigma (i)},\sum_{i=1}^{m}\omega_{i}v_{\sigma (i)}\right) \end{array}$$

*where* $\omega ={\left(\omega_{1},\omega_{2},...,\omega_{m}\right)}^{T}$ *is an associated weight vector with* $\sum_{i=1}^{m}\omega_{i}=1$ *and* $\omega_{i}\in [0,1]$ *(i = 1, 2, …, m),* $I_{i}$ *is the value of an order inducing variable (i = 1, 2, …, m), and* σ *is a permutation of {1, 2, …, m} such that* $I_{\sigma (i)}\leq I_{\sigma (i+1)}$ *for each i = 1, 2, …, m − 1.*

As $\alpha_{i}=(\mu_{i},v_{i})$ is an IFN, we have $0\leq \mu_{i}\leq 1,0\leq v_{i}\leq 1$ and $\mu_{i}+v_{i}\leq 1$. Thus, one can obtain $0\leq \sum_{i=1}^{m}\omega_{i}\mu_{\sigma (i)}\leq 1,0\leq \sum_{i=1}^{m}\omega_{i}v_{\sigma (i)}\leq 1$ and $\sum_{i=1}^{m}\omega_{i}\mu_{\sigma (i)}+\sum_{i=1}^{m}\omega_{i}v_{\sigma (i)}=\sum_{i=1}^{m}\omega_{i}(\mu_{\sigma (i)}+v_{\sigma (i)})\leq 1$. Therefore, the aggregated value by using the IIOWA operator remains an IFN. Obviously, if $\mu_{i}=v_{i}$ for all *i* = 1, 2, …, m, one has $\sum_{i=1}^{m}\omega_{i}\mu_{\sigma (i)}=\sum_{i=1}^{m}\omega_{i}v_{\sigma (i)}$.

**Theorem 4.**  The IIOWA operator defined in (15) is idempotent, commutative, bounded, and monotonic with respect to the order based on score and accuracy functions.

**Proof.**  The idempotence and commutativity of the IIOWA operator can be directly obtained from Definition 4.

Let $\alpha_{\min}=(\mu_{\min},v_{\min})=\min\{\alpha_{i}|i=1,2...,m\}$ and $\alpha_{\max}=(\mu_{\max},v_{\max})=\max\{\alpha_{i}|i=1,2...,m\}$. According to the prioritized comparison method for any two IFNs in Section 2, one has

(i) $S(\alpha_{\min})=\mu_{\min}-v_{\min}<S(\alpha_{i})=\mu_{i}-v_{i}$ or $\mu_{\min}-v_{\min}=\mu_{i}-v_{i}\;\mathrm{and}\;\mu_{\min}+v_{\min}\leq \mu_{i}+v_{i}.$
(ii) $S(\alpha_{i})=\mu_{i}-v_{i}<S(\alpha_{\max})=\mu_{\max}-v_{\max}$ or $\mu_{i}-v_{i}=\mu_{\max}-v_{\max}\;\mathrm{and}\;\mu_{i}+v_{i}\leq \mu_{\max}+v_{\max}$.
  Thus, one can obtain
(iii) $S(\alpha_{\min})=\mu_{\min}-v_{\min}<\sum_{i=1}^{m}\omega_{i}(\mu_{\sigma (i)}-v_{\sigma (i)})=S(IIOWA_{\omega }\left(<I_{1},\alpha_{1}>,<I_{2},\alpha_{2}>,...,<I_{m},\alpha_{m}>\right))$
  or
  $S(\alpha_{\min})=S(IIOWA_{\omega }\left(<I_{1},\alpha_{1}>,<I_{2},\alpha_{2}>,...,<I_{m},\alpha_{m}>\right))\;\mathrm{and}\;\mu_{\min}+v_{\min}\leq \sum_{i=1}^{m}\omega_{i}(\mu_{\sigma (i)}+v_{\sigma (i)})$.
(iv) $\sum_{i=1}^{m}\omega_{i}(\mu_{\sigma (i)}-v_{\sigma (i)})=S(IIOWA_{\omega }\left(<I_{1},\alpha_{1}>,<I_{2},\alpha_{2}>,...,<I_{m},\alpha_{m}>\right))<S(\alpha_{\max})=\mu_{\max}-v_{\max}$
  or
  $S(IIOWA_{\omega }\left(<I_{1},\alpha_{1}>,<I_{2},\alpha_{2}>,...,<I_{m},\alpha_{m}>\right))=S(\alpha_{\max})\;\mathrm{and}\;\sum_{i=1}^{m}\omega_{i}(\mu_{\sigma (i)}+v_{\sigma (i)})\leq \mu_{\max}+v_{\max}$.

Therefore, $\alpha_{\min}\leq IIOWA_{\omega }\left(<I_{1},\alpha_{1}>,<I_{2},\alpha_{2}>,...,<I_{m},\alpha_{m}>\right)\leq \alpha_{\max},$ verifying the boundedness of the IIOWA operator in terms of the score and accuracy functions.

For the monotonicity, let $\alpha_{i}\leq \alpha_{i}^{\prime }$ for all *i* (*i* = 1, 2, …, *m*), where $\alpha_{i}^{\prime }=(\mu_{i}^{\prime },v_{i}^{\prime })$, then as per the comparison method for two IFNs, we have

$$S(\alpha_{i})=\mu_{i}-v_{i}<S(\alpha_{i}^{\prime })=\mu_{i}^{\prime }-v_{i}^{\prime }\;\mathrm{or}\;\mu_{i}-v_{i}=\mu_{i}^{\prime }-v_{i}^{\prime }\;\mathrm{and}\;\mu_{i}+v_{i}\leq \mu_{i}^{\prime }+v_{i}^{\prime }$$

By applying the IIOWA formula in Definition 4, one has

$$\sum_{i=1}^{m}\omega_{i}\mu_{\sigma (i)}-\sum_{i=1}^{m}\omega_{i}v_{\sigma (i)}<\sum_{i=1}^{m}\omega_{i}\mu_{\sigma (i)}^{\prime }-\sum_{i=1}^{m}\omega_{i}v_{\sigma (i)}^{\prime }\;\mathrm{or}$$

$$\sum_{i=1}^{m}\omega_{i}\mu_{\sigma (i)}-\sum_{i=1}^{m}\omega_{i}v_{\sigma (i)}=\sum_{i=1}^{m}\omega_{i}\mu_{\sigma (i)}^{\prime }-\sum_{i=1}^{m}\omega_{i}v_{\sigma (i)}^{\prime }\;\mathrm{and}\;\sum_{i=1}^{m}\omega_{i}\mu_{\sigma (i)}+\sum_{i=1}^{m}\omega_{i}v_{\sigma (i)}\leq \sum_{i=1}^{m}\omega_{i}\mu_{\sigma (i)}^{\prime }+\sum_{i=1}^{m}\omega_{i}v_{\sigma (i)}^{\prime }$$

As per the comparison approach of IFNs, one can get $\left(\sum_{i=1}^{m}\omega_{i}\mu_{\sigma (i)},\sum_{i=1}^{m}\omega_{i}v_{\sigma (i)}\right)\leq \left(\sum_{i=1}^{m}\omega_{i}\mu_{\sigma (i)}^{\prime },\sum_{i=1}^{m}\omega_{i}v_{\sigma (i)}^{\prime }\right)$. It follows from (15) that

$$IIOWA_{\omega }\left(<I_{1},\alpha_{1}>,<I_{2},\alpha_{2}>,...,<I_{m},\alpha_{m}>\right)\leq IIOWA_{\omega }\left(<I_{1},\alpha_{1}^{\prime }>,<I_{2},\alpha_{2}^{\prime }>,...,<I_{m},\alpha_{m}^{\prime }>\right)$$.

The proof of Theorem 4 is thus completed.  ☐

As per (15), it is easy to prove the following theorem.

**Theorem 5.**  *Let* $\alpha_{i}=(\mu_{i},v_{i})$ *(i = 1, 2, …, m) be m IFNs, then*

(16) $$IIOWA_{\omega }\left(<I_{1},\alpha_{1}^{C}>,<I_{2},\alpha_{2}^{C}>,...,<I_{m},\alpha_{m}^{C}>\right)=\left(\sum_{i=1}^{m}\omega_{i}v_{\sigma (i)},\sum_{i=1}^{m}\omega_{i}\mu_{\sigma (i)}\right)$$

*where* $\alpha_{i}^{C}$ *is the complement of* $\alpha_{i}$*, i.e.,* $\alpha_{i}^{C}=(v_{i},\mu_{i})$ *for each* i=1,2,...,m.

Theorem 5 indicates that for *m* IFNs, we have $IIOWA_{\omega }\left(<I_{1},\alpha_{1}^{C}>,<I_{2},\alpha_{2}^{C}>,...,<I_{m},\alpha_{m}^{C}>\right)=$ ${\left(IIOWA_{\omega }\left(<I_{1},\alpha_{1}>,<I_{2},\alpha_{2}>,...,<I_{m},\alpha_{m}>\right)\right)}^{C}$, implying that the complement property will be maintained by using the IIOWA operator to aggregate m IFNs into an IFN. It is noted that this property does not hold for the aggregation operator I-IFOWA introduced by Wei [28].

Apparently, the order inducing variable follows an ascending order in Definition 4, which can be conveniently reversed to obtain a descending IIOWA (DIIOWA) operator.

### 4.2. Properties of IIOWA Aggregation of Intuitionistic Preference Relations

Next, we shall investigate the properties of the aggregation result when the IIOWA operator is applied to aggregate intuitionistic preference relations.

**Theorem 6.**  *Let* ${\tilde{R}}^{k}={\left({\tilde{r}}_{ij}^{k}\right)}_{n\times n}$ *with* ${\tilde{r}}_{ij}^{k}=(\mu_{ij}^{k},v_{ij}^{k})$ *(k = 1, 2, …, m) be m intuitionistic preference relations, and* $I_{k}$ *(k = 1, 2, …, m) be m values of the order inducing variable, then the aggregation* ${\tilde{R}}^{G}={\left({\tilde{r}}_{ij}^{G}\right)}_{n\times n}={\left(\left(\mu_{ij}^{G},v_{ij}^{G}\right)\right)}_{n\times n}={\left(IIOWA_{\omega }(<I_{1},{\tilde{r}}_{ij}^{1}>,<I_{2},{\tilde{r}}_{ij}^{2}>,...,<I_{m},{\tilde{r}}_{ij}^{m}>)\right)}_{n\times n}$*is also an intuitionistic preference relation.*

**Proof.**  As ${\tilde{R}}^{k}={\left({\tilde{r}}_{ij}^{k}\right)}_{n\times n}={\left(\left(\mu_{ij}^{k},v_{ij}^{k}\right)\right)}_{n\times n}$ is an intuitionistic preference relation, as per (5), we have $\mu_{ij}^{k}=v_{ji}^{k},v_{ij}^{k}=\mu_{ji}^{k},\mu_{ii}^{k}=v_{ii}^{k}=0.5$ ∀i,j=1,2,...,n,k=1,2,...,m.

According to Definition 4, one has $\mu_{ij}^{G}=\sum_{k=1}^{m}\omega_{k}\mu_{ij}^{\sigma (k)}$ and $v_{ij}^{G}=\sum_{k=1}^{m}\omega_{k}v_{ij}^{\sigma (k)}$. Thus, $\mu_{ij}^{G}=\sum_{k=1}^{m}\omega_{k}\mu_{ij}^{\sigma (k)}=\sum_{k=1}^{m}\omega_{k}v_{ji}^{\sigma (k)}=v_{ji}^{G},v_{ij}^{G}=\sum_{k=1}^{m}\omega_{k}v_{ij}^{\sigma (k)}=\sum_{k=1}^{m}\omega_{k}\mu_{ji}^{\sigma (k)}=\mu_{ji}^{G},\mu_{ii}^{G}=\sum_{k=1}^{m}\omega_{k}\mu_{ii}^{\sigma (k)}=0.5$ and $v_{ii}^{G}=\sum_{k=1}^{m}\omega_{k}v_{ii}^{\sigma (k)}=0.5$. Therefore, ${\tilde{R}}^{G}$ is an intuitionistic preference relation. ☐

**Theorem 7.**  *If* ${\tilde{R}}^{k}={\left({\tilde{r}}_{ij}^{k}\right)}_{n\times n}={\left(\left(\mu_{ij}^{k},v_{ij}^{k}\right)\right)}_{n\times n}$ *is an additive consistent intuitionistic preference relation for each k = 1, 2, …, m, then the aggregation* ${\tilde{R}}^{G}={\left({\tilde{r}}_{ij}^{G}\right)}_{n\times n}=$ ${\left(\left(\mu_{ij}^{G},v_{ij}^{G}\right)\right)}_{n\times n}=$ ${\left(IIOWA_{\omega }(<I_{1},{\tilde{r}}_{ij}^{1}>,<I_{2},{\tilde{r}}_{ij}^{2}>,...,<I_{m},{\tilde{r}}_{ij}^{m}>)\right)}_{n\times n}$ *is also additive consistent.*

**Proof.**  As per (2) and (15), we have

$$S({\tilde{r}}_{ij}^{G})=\mu_{ij}^{G}-v_{ij}^{G}=\sum_{k=1}^{m}\omega_{k}\mu_{ij}^{\sigma (k)}-\sum_{k=1}^{m}\omega_{k}v_{ij}^{\sigma (k)}=\sum_{k=1}^{m}\omega_{k}\left(\mu_{ij}^{\sigma (k)}-v_{ij}^{\sigma (k)}\right)=\sum_{k=1}^{m}\omega_{k}S({\tilde{r}}_{ij}^{\sigma (k)})$$

and

$$\begin{array}{l} S({\tilde{r}}_{il}^{G})-S({\tilde{r}}_{jl}^{G})=\mu_{il}^{G}-v_{il}^{G}-(\mu_{jl}^{G}-v_{jl}^{G})=\sum_{k=1}^{m}\omega_{k}\mu_{il}^{\sigma (k)}-\sum_{k=1}^{m}\omega_{k}v_{il}^{\sigma (k)}-\sum_{k=1}^{m}\omega_{k}\mu_{jl}^{\sigma (k)}+\sum_{k=1}^{m}\omega_{k}v_{jl}^{\sigma (k)}\\ =\sum_{k=1}^{m}\omega_{k}\left(\mu_{il}^{\sigma (k)}-v_{il}^{\sigma (k)}\right)-\sum_{k=1}^{m}\omega_{k}\left(\mu_{jl}^{\sigma (k)}-v_{jl}^{\sigma (k)}\right)=\sum_{k=1}^{m}\omega_{k}\left(S({\tilde{r}}_{il}^{\sigma (k)})-S({\tilde{r}}_{jl}^{\sigma (k)})\right) \end{array}$$

On the other hand, as ${\tilde{R}}^{k}$ is additive consistent, it follows from Lemma 1 that

$$S({\tilde{r}}_{ij}^{k})=S({\tilde{r}}_{il}^{k})-S({\tilde{r}}_{jl}^{k})\;\text{for all}\;i,j,l=1,2,\ldots ,n,k=1,2,\ldots ,m.$$

Therefore, $S({\tilde{r}}_{il}^{G})-S({\tilde{r}}_{jl}^{G})=\sum_{k=1}^{m}\omega_{k}\left(S({\tilde{r}}_{il}^{\sigma (k)})-S({\tilde{r}}_{jl}^{\sigma (k)})\right)=\sum_{k=1}^{m}\omega_{k}S({\tilde{r}}_{ij}^{\sigma (k)})=S({\tilde{r}}_{ij}^{G})$ for all *i*, *j*, *l =* 1, 2,…, *n*. By Lemma 1, ${\tilde{R}}^{G}$ is additive consistent. ☐

Theorem 6 indicates that an intuitionistic preference relation ${\tilde{R}}^{G}$ will be obtained by applying the IIOWA operator to aggregate individual intuitionistic judgments ${\tilde{R}}^{k}$ (*k* = 1, 2, …, *m*). Theorem 7 further confirms that the resulting ${\tilde{R}}^{G}$ is additive consistent provided that all individual intuitionistic preference relations are also consistent.

### 4.3. An IIOWA-Aggregation-Based Procedure for Group Decision with Intuitionistic Preference Relations

Consider a group decision problem with *m* DMs, $D=\{d_{1},d_{2},...,d_{m}\}$. Each DM $d_{k}\in D$ (k=1,2,...,m) furnishes its assessment on an alternative set $X=\{x_{1},x_{2},...,x_{n}\}$ as an intuitionistic preference relation ${\tilde{R}}^{k}={\left({\tilde{r}}_{ij}^{k}\right)}_{n\times n}={\left(\left(\mu_{ij}^{k},v_{ij}^{k}\right)\right)}_{n\times n}$.

If a given intuitionistic preference relation ${\tilde{R}}^{k}$ is not additive consistent, by employing the proposed rectification method in Section 3, a consistent intuitionistic preference relation ${\hat{\tilde{R}}}^{\prime k}={\left({\hat{\tilde{r}}}_{ij}^{\prime k}\right)}_{n\times n}={\left(\left({\hat{\mu }}_{ij}^{\prime k},{\hat{v}}_{ij}^{\prime k}\right)\right)}_{n\times n}$ can be obtained for DM *d_k_*. Based on ${\hat{\tilde{R}}}^{\prime k}$ (k=1,2,...,m), the next stage in the solution process for a GDM problem is to derive a consistent group intuitionistic preference relation by a certain aggregation procedure. To obtain a reasonable result, the aggregation operator should properly account for the importance degree of each DM’s rectified ${\hat{\tilde{R}}}^{\prime k}$ (*k* = 1, 2, …, *m*).

In this model, the importance degree of each DM *d_k_* (*k* = 1, 2, …, *m*) is assumed to be completely unknown. As such, a rational way in the aggregation process is to associate the importance degree with the deviation between the rectified consistent intuitionistic preference relation and the original judgment matrix. In order to measure the importance degree of a DM’s ${\hat{\tilde{R}}}^{\prime k}$, Wang and Li [9] introduced the following definition.

**Definition 5.**  *Let* $\tilde{R}={\left({\tilde{r}}_{ij}\right)}_{n\times n}={\left(\left(\mu_{ij},v_{ij}\right)\right)}_{n\times n}$ *and* ${\tilde{R}}^{\prime }={\left({\tilde{r}}_{ij}^{\prime }\right)}_{n\times n}={\left(\left(\mu_{ij}^{\prime },v_{ij}^{\prime }\right)\right)}_{n\times n}$ *be any two intuitionistic preference relations, the mean absolute deviation (MAD) between* $\tilde{R}$ *and* ${\tilde{R}}^{\prime }$ *is defined as:*

(17) $$MAD(\tilde{R},{\tilde{R}}^{\prime })=\frac{1}{2n(n-1)}\sum_{i=1}^{n}\sum_{j=1,j\neq i}^{n}\left(\left|\mu_{ij}-\mu_{ij}^{\prime }\right|+\left|v_{ij}-v_{ij}^{\prime }\right|\right)$$

Obviously, $0\leq MAD(\tilde{R},{\tilde{R}}^{\prime })\leq 1$ and $MAD(\tilde{R},{\tilde{R}}^{\prime })=MAD({\tilde{R}}^{\prime },\tilde{R})$. The smaller the value of $MAD(\tilde{R},{\tilde{R}}^{\prime })$, the closer $\tilde{R}$ is to ${\tilde{R}}^{\prime }$. Especially, if $MAD(\tilde{R},{\tilde{R}}^{\prime })=0$, $\tilde{R}$ = ${\tilde{R}}^{\prime }$.

By applying (17) to the rectified ${\hat{\tilde{R}}}^{\prime k}$ and the original ${\tilde{R}}^{k}$ for each *k* = 1, 2, …, *m*, we obtain *m* MAD values $\left\{MAD({\hat{\tilde{R}}}^{\prime k},{\tilde{R}}^{k})|k=1,2...,m\right\}$. The smaller the value of $MAD({\hat{\tilde{R}}}^{\prime k},{\tilde{R}}^{k})$, the closer the rectified ${\hat{\tilde{R}}}^{\prime k}$ is to the original intuitionistic judgment ${\tilde{R}}^{k}$. It is sensible to assign a higher importance level for ${\hat{\tilde{R}}}^{\prime k}$ in the aggregation process. Thus, $MAD({\hat{\tilde{R}}}^{\prime k},{\tilde{R}}^{k})$ will be adopted as the order inducing variable in aggregating ${\hat{\tilde{R}}}^{\prime k}$.

Once the order inducing variable is determined, a natural issue in the aggregation of $\left\{{\hat{\tilde{R}}}^{\prime 1},{\hat{\tilde{R}}}^{\prime 2},...,{\hat{\tilde{R}}}^{\prime m}\right\}$ by using the IIOWA operator is to calculate the weight vector associated with the order inducing variable. A number of approaches [33] have been developed for determining the associated weights. This paper adopts the following formula that is initially proposed by Yager [34]:

(18) $$\omega_{k}=\frac{{\left(1-MAD({\hat{\tilde{R}}}^{\prime \sigma (k)},{\tilde{R}}^{\sigma (k)})\right)}^{\lambda }}{\sum_{k=1}^{m}{\left(1-MAD({\hat{\tilde{R}}}^{\prime \sigma (k)},{\tilde{R}}^{\sigma (k)})\right)}^{\lambda }}\;k=1,2,\ldots ,m$$

where λ∈(0,+∞).

In the context of the inducing variable here, it is desirable that a higher weight is assigned to a lower MAD value. Without loss of generality, λ=2 is used to determine the associated weights.

Based on the weights determined by (18), a group intuitionistic preference relation ${\tilde{R}}^{\prime G}={\left({\tilde{r}}_{ij}^{\prime G}\right)}_{n\times n}$ with ${\tilde{r}}_{ij}^{\prime G}=(\mu_{ij}^{\prime G},v_{ij}^{\prime G})$ can be derived from ${\hat{\tilde{R}}}^{\prime k}$ (*k* = 1, 2, …, *m*) by employing the IIOWA operator. According to Theorem 7, ${\tilde{R}}^{\prime G}$ is additive consistent.

The last stage in the solution process is to obtain a ranking of all alternatives or select the best one(s) based on the aggregated ${\tilde{R}}^{\prime G}$. To facilitate the ranking process, define:

(19) $$s_{i}=\frac{1}{n}\sum_{l=1}^{n}S({\tilde{r}}_{il}^{\prime G})\;i=1,2,\ldots ,n$$

**Theorem 8.**  *Let* $s_{i}$ *(i = 1, 2, …, n) be defined by (19), if* $S({\tilde{r}}_{ij}^{\prime G})\geq 0$ *(*i,j∈{1,2,...,n}*), then* $s_{i}\geq s_{j}.$

**Proof.**  As ${\tilde{R}}^{\prime G}$ is an additive consistent intuitionistic preference relation, it follows from (8) that $S({\tilde{r}}_{ij}^{\prime G})=S({\tilde{r}}_{il}^{\prime G})-S({\tilde{r}}_{jl}^{\prime G}),i,j,l=1,2,...,n$.

Plugging in (19), one has $s_{i}-s_{j}=\frac{1}{n}\left(\sum_{l=1}^{n}\left(S({\tilde{r}}_{il}^{\prime G})-S({\tilde{r}}_{jl}^{\prime G})\right)\right)\;=S({\tilde{r}}_{ij}^{\prime G})$.

If $S({\tilde{r}}_{ij}^{\prime G})\geq 0$, it immediately follows $s_{i}\geq s_{j}$. This proves Theorem 8. ☐

Theorem 8 indicates that a ranking order of alternatives as per a descending order of $s_{i}$ (*i* = 1, 2, …, *n*) is consistent with the score-function-based ranking derived from the aggregated preference values in ${\tilde{R}}^{\prime G}$.

Based on the aforesaid analyses, a solution procedure is summarized below for GDM with intuitionistic preference relations.

Step 1. For each intuitionistic preference relation ${\tilde{R}}^{k}$ furnished by DM $d_{k}\in D$ (k=1,2,...,m), use Lemma 1 to determine if it is additive consistent. If ${\tilde{R}}^{k}$ is consistent, ${\hat{\tilde{R}}}^{\prime k}={\tilde{R}}^{k}$. Otherwise, employ the rectification process in Section 3 to construct an additive consistent intuitionistic preference relation ${\hat{\tilde{R}}}^{\prime k}$ for each inconsistent ${\tilde{R}}^{k}$.

Step 2. Calculate MAD value $MAD({\hat{\tilde{R}}}^{\prime k},{\tilde{R}}^{k})$ between the constructed consistent intuitionistic preference relation ${\hat{\tilde{R}}}^{\prime k}$ and the original judgment matrix ${\tilde{R}}^{k}$ for each k=1,2,...,m as per (17).

Step 3. Determine the associated weights $\omega_{k}$ (*k* = 1, 2, …, *m*) by plugging the values of $MAD({\tilde{R}}^{\sigma (k)},{\hat{\tilde{R}}}^{\prime \sigma (k)})$ into (18) with λ=2.

Step 4. Use the IIOWA operator to aggregate all ${\hat{\tilde{R}}}^{\prime k}$(*k* = 1, 2, …, *m*) into a collective consistent intuitionistic preference relation ${\tilde{R}}^{\prime G}={\left({\tilde{r}}_{ij}^{\prime G}\right)}_{n\times n}$ with ${\tilde{r}}_{ij}^{\prime G}=(\mu_{ij}^{\prime G},v_{ij}^{\prime G})$, where ${\tilde{r}}_{ij}^{\prime G}=IIOWA_{\omega }(<MAD({\hat{\tilde{R}}}^{\prime 1},{\tilde{R}}^{1}),{\tilde{r}}_{ij}^{\prime 1}>,<MAD({\hat{\tilde{R}}}^{\prime 2},{\tilde{R}}^{2}),{\tilde{r}}_{ij}^{\prime 2}>,...,<MAD({\hat{\tilde{R}}}^{\prime m},{\tilde{R}}^{m}),{\tilde{r}}_{ij}^{\prime m}>)$ (*i*, *j* = 1, 2, …, *n*), i.e., $\mu_{ij}^{\prime G}=\sum_{k=1}^{m}\omega_{k}{\hat{\mu }}_{ij}^{\prime \sigma (k)}$ and $v_{ij}^{\prime G}=\sum_{k=1}^{m}\omega_{k}{\hat{v}}_{ij}^{\prime \sigma (k)}$ (*i, j* =1, 2, …, *n*).

Step 5. Obtain the ranking value $s_{i}$ for alternative $x_{i}\in X$ (*i* = 1, 2, …, *n*) as per (19).

Step 6. Rank alternatives and select the best one(s) according to a decreasing order of $s_{i}$ (*i* =1, 2, …, *n*).

## 5. An Example of Low Carbon Supplier Selection

This section applies the proposed procedure to GDM concerning low carbon supplier selection where the DMs’ judgment information is furnished as intuitionistic preference relations (adapted from Theißen and Spinler [35]).

To select an appropriate low carbon supplier for a manufacturer, a committee consisting of three members, $d_{1},d_{2}$, and $d_{3}$, is convened and the members are from the procurement, production, and finance departments. These representatives offer their assessments on four potential suppliers $x_{1},x_{2},x_{3}$, and $x_{4}$ based on a set of criteria accounting for low carbon technology, cost, and capacity. It is assumed that each DM $d_{k}$ (*k* = 1, 2, 3) gives its pairwise comparison results over the four suppliers as the following intuitionistic preference relations ${\tilde{R}}^{k}={\left({\tilde{r}}_{ij}^{k}\right)}_{4\times 4}$ with ${\tilde{r}}_{ij}^{k}=(\mu_{ij}^{k},v_{ij}^{k})$:

$${\tilde{R}}^{1}=\left[\begin{array}{cccc} \left(0.5,0.5\right) & \left(0.4,0.5\right) & \left(0.6,0.3\right) & \left(0.3,0.5\right)\\ \left(0.5,0.4\right) & \left(0.5,0.5\right) & \left(0.5,0.3\right) & \left(0.6,0.3\right)\\ \left(0.3,0.6\right) & \left(0.3,0.5\right) & \left(0.5,0.5\right) & \left(0.6,0.2\right)\\ \left(0.5,0.3\right) & \left(0.3,0.6\right) & \left(0.2,0.6\right) & \left(0.5,0.5\right) \end{array}\right]$$

$${\tilde{R}}^{2}=\left[\begin{array}{cccc} \left(0.5,0.5\right) & \left(0.3,0.4\right) & \left(0.9,0.0\right) & \left(0.9,0.0\right)\\ \left(0.4,0.3\right) & \left(0.5,0.5\right) & \left(0.2,0.5\right) & \left(0.95,0.0\right)\\ \left(0.0,0.9\right) & \left(0.5,0.2\right) & \left(0.5,0.5\right) & \left(0.95,0.0\right)\\ \left(0.0,0.9\right) & \left(0.0,0.95\right) & \left(0.0,0.95\right) & \left(0.5,0.5\right) \end{array}\right]$$

$${\tilde{R}}^{3}=\left[\begin{array}{cccc} \left(0.5,0.5\right) & \left(0.1,0.6\right) & \left(0.7,0.0\right) & \left(0.3,0.2\right)\\ \left(0.6,0.1\right) & \left(0.5,0.5\right) & \left(0.8,0.2\right) & \left(0.9,0.0\right)\\ \left(0.0,0.7\right) & \left(0.2,0.8\right) & \left(0.5,0.5\right) & \left(0.8,0.1\right)\\ \left(0.2,0.3\right) & \left(0,0.9\right) & \left(0.1,0.8\right) & \left(0.5,0.5\right) \end{array}\right]$$

It is easy to verify that these three intuitionistic preferences are not additive consistent based on Definition 2. As such, the rectification process in Section 3 has to be carried out. As per (9) and (10), the following three transformation matrices are obtained:

$${\hat{\tilde{R}}}^{1}=\left[\begin{array}{cccc} \left(0.5,0.5\right) & \left(0.3750,0.5250\right) & \left(0.4625,0.4375\right) & \left(0.4625,0.3375\right)\\ \left(0.5250,0.3750\right) & \left(0.5,0.5\right) & \left(0.4875,0.3125\right) & \left(0.5875,0.3125\right)\\ \left(0.4375,0.4625\right) & \left(0.3125,0.4875\right) & \left(0.5,0.5\right) & \left(0.4500,0.3500\right)\\ \left(0.3375,0.4625\right) & \left(0.3125,0.5875\right) & \left(0.3500,0.4500\right) & \left(0.5,0.5\right) \end{array}\right]$$

$${\hat{\tilde{R}}}^{2}=\left[\begin{array}{cccc} \left(0.5,0.5\right) & \left(0.46875,0.23125\right) & \left(0.61875,0.28125\right) & \left(1.0125,-0.1125\right)\\ \left(0.23125,0.46875\right) & \left(0.5,0.5\right) & \left(0.40000,0.30000\right) & \left(0.91875,0.03125\right)\\ \left(0.28125,0.61875\right) & \left(0.30000,0.40000\right) & \left(0.5,0.5\right) & \left(0.86875,0.08125\right)\\ \left(-0.1125,1.0125\right) & \left(0.03125,0.91875\right) & \left(0.08125,0.86875\right) & \left(0.5,0.5\right) \end{array}\right]$$

$${\hat{\tilde{R}}}^{3}=\left[\begin{array}{cccc} \left(0.5,0.5\right) & \left(0.1375,0.5625\right) & \left(0.4625,0.2375\right) & \left(0.5000,0.0000\right)\\ \left(0.5625,0.1375\right) & \left(0.5,0.5\right) & \left(0.8250,0.1750\right) & \left(0.9125,-0.0125\right)\\ \left(0.2375,0.4625\right) & \left(0.1750,0.8250\right) & \left(0.5,0.5\right) & \left(0.5875,0.3125\right)\\ \left(0.0000,0.5000\right) & \left(-0.0125,0.9125\right) & \left(0.3125,0.5875\right) & \left(0.5,0.5\right) \end{array}\right]$$

In ${\hat{\tilde{R}}}^{1}$, $0<{\hat{\mu }}_{ij}<1$ and $0<{\hat{v}}_{ij}<1$ for all i,j=1,2,3,4. In ${\hat{\tilde{R}}}^{2}$, ${\hat{\mu }}_{41}<0$ and ${\hat{v}}_{41}>1$ (correspondingly, ${\hat{\mu }}_{14}>1$ and ${\hat{v}}_{14}<0$). In ${\hat{\tilde{R}}}^{3}$, ${\hat{\mu }}_{42}<0$ (correspondingly, ${\hat{v}}_{24}<0$). By (11), the *d* values for ${\hat{\tilde{R}}}^{1}$, ${\hat{\tilde{R}}}^{2}$, and ${\hat{\tilde{R}}}^{3}$ are 0, 0.1125, and 0.0125, respectively.

According to (13), we obtain three additively consistent intuitionistic preference relations as follows.

$${\hat{\tilde{R}}}^{\prime 1}={\hat{\tilde{R}}}^{1}=\left[\begin{array}{cccc} \left(0.5,0.5\right) & \left(0.3750,0.5250\right) & \left(0.4625,0.4375\right) & \left(0.4625,0.3375\right)\\ \left(0.5250,0.3750\right) & \left(0.5,0.5\right) & \left(0.4875,0.3125\right) & \left(0.5875,0.3125\right)\\ \left(0.4375,0.4625\right) & \left(0.3125,0.4875\right) & \left(0.5,0.5\right) & \left(0.4500,0.3500\right)\\ \left(0.3375,0.4625\right) & \left(0.3125,0.5875\right) & \left(0.3500,0.4500\right) & \left(0.5,0.5\right) \end{array}\right]$$

$${\hat{\tilde{R}}}^{\prime 2}=\left[\begin{array}{cccc} \left(0.5,0.5\right) & \left(0.47449,0.28061\right) & \left(0.59694,0.32143\right) & \left(0.91837,0.00000\right)\\ \left(0.28061,0.47449\right) & \left(0.5,0.5\right) & \left(0.41837,0.33673\right) & \left(0.84184,0.11735\right)\\ \left(0.32143,0.59694\right) & \left(0.33673,0.41837\right) & \left(0.5,0.5\right) & \left(0.80102,0.15816\right)\\ \left(0.00000,0.91837\right) & \left(0.11735,0.84184\right) & \left(0.15816,0.80102\right) & \left(0.5,0.5\right) \end{array}\right]$$

$${\hat{\tilde{R}}}^{\prime 3}=\left[\begin{array}{cccc} \left(0.5,0.5\right) & \left(0.14634,0.56098\right) & \left(0.46341,0.24390\right) & \left(0.50000,0.01220\right)\\ \left(0.56098,0.14634\right) & \left(0.5,0.5\right) & \left(0.81707,0.18293\right) & \left(0.90244,0.00000\right)\\ \left(0.24390,0.46341\right) & \left(0.18293,0.81707\right) & \left(0.5,0.5\right) & \left(0.58537,0.31707\right)\\ \left(0.01220,0.50000\right) & \left(0.00000,0.90244\right) & \left(0.31707,0.58537\right) & \left(0.5,0.5\right) \end{array}\right]$$

One can easily verify that the hesitancy degrees of IFNs in ${\hat{\tilde{R}}}^{\prime 1}$ are equal to those of the corresponding IFNs in the original intuitionistic preference relation ${\tilde{R}}^{1}$. For the constructed consistent intuitionistic preference relations ${\hat{\tilde{R}}}^{\prime 2}$ and ${\hat{\tilde{R}}}^{\prime 3}$, one can see that the hesitancy degrees of the original preferences in ${\tilde{R}}_{2}$ and ${\tilde{R}}_{3}$ are reduced by a factor of 1/1.225 and 1/1.025, respectively.

By using (17), the MAD values $MAD({\hat{\tilde{R}}}^{\prime k},{\tilde{R}}^{k})$ (*k* = 1, 2, 3) between the constructed consistent intuitionistic preference relations ${\hat{\tilde{R}}}^{\prime k}$ and the original judgment matrix ${\tilde{R}}^{k}$ are determined as

$$MAD({\hat{\tilde{R}}}^{\prime 1},{\tilde{R}}^{1})=0.08333,\;MAD({\hat{\tilde{R}}}^{\prime 2},{\tilde{R}}^{2})=0.15425,\;MAD({\hat{\tilde{R}}}^{\prime 3},{\tilde{R}}^{3})=0.11849$$

As $MAD({\hat{\tilde{R}}}^{\prime 1},{\tilde{R}}^{1})<MAD({\hat{\tilde{R}}}^{\prime 3},{\tilde{R}}^{3})<MAD({\hat{\tilde{R}}}^{\prime 2},{\tilde{R}}^{2})$, as per Definition 4, if the MAD is adopted as the order inducing variable, then a permutation of {1, 2, 3} can be obtained as {1, 3, 2}, i.e., σ(1)=1,σ(2)=3 and σ(3)=2. Therefore, based on (18) with λ=2, the associated DM weights of the IIOWA operator are determined as $\omega_{1}=0.3602$, $\omega_{2}=0.3331$, and $\omega_{3}=0.3067$.

By employing the IIOWA operator with the associated weight vector ${\left(\omega_{1},\omega_{2},\omega_{3}\right)}^{T}$ and the permutation σ, the collective consistent intuitionistic preference relation ${\tilde{R}}^{\prime G}={\left({\tilde{r}}_{ij}^{\prime G}\right)}_{n\times n}$ is derived as

$${\hat{\tilde{R}}}^{\prime G}=\left[\begin{array}{cccc} \left(0.5,0.5\right) & \left(0.32935,0.46203\right) & \left(0.50404,0.33741\right) & \left(0.61481,0.12563\right)\\ \left(0.46203,0.32935\right) & \left(0.5,0.5\right) & \left(0.57608,0.27677\right) & \left(0.77041,0.14855\right)\\ \left(0.33741,0.50404\right) & \left(0.27677,0.57608\right) & \left(0.5,0.5\right) & \left(0.60275,0.28019\right)\\ \left(0.12563,0.61481\right) & \left(0.14855,0.77041\right) & \left(0.28019,0.60275\right) & \left(0.5,0.5\right) \end{array}\right]$$

As per (19), the ranking values for all alternatives are determined

$$s_{1}=0.13078,s_{2}=0.26346,s_{3}=-0.03585,s_{4}=-0.3584$$

Since $s_{2}>s_{1}>s_{3}>s_{4}$, a full ranking of the four suppliers is obtained as $x_{2}≻x_{1}≻x_{3}≻x_{4}$.

## 6. Conclusions

Consistency and aggregation are two critical issues in GDM with intuitionistic preference relations. This paper puts forward a three-stage framework to handle GDM problems with intuitionistic preference relations and applies it to solve low carbon supplier selection problems. Based on the additive consistency definition proposed by Wang [19], the first stage is concerned with rectifying the original inconsistent intuitionistic preference relations furnished by the DMs. In the aggregation stage, a new intuitionistic fuzzy aggregation operator, the so-called IIOWA, is developed to aggregate the rectified consistent intuitionistic preference relations. This aggregation operator adopts the MAD value between the original and rectified intuitionistic preference relations as an order inducing variable. Based on the aggregated consistent intuitionistic preference relation, an overall ranking function is defined to rank alternatives or select the best one(s).

Significant research opportunities remain open along this line of research. For instance, it is unclear how to handle missing values in the intuitionistic preference relations furnished by the DMs. In addition, the current research rectifies inconsistent intuitionistic preference relations to completely additive consistent. However, sometimes complete consistency may not be necessary as long as inconsistency is controlled to within an acceptable level. It would be interesting to examine how the current framework can be adapted to handle these extensions.
